# Supplementary material for: Knowledge reuse in software projects: Retrieving software development Q&A posts based on project task similarity
Source: PLoS One. 2020 Dec 17;15(12):e0243852. doi: 10.1371/journal.pone.0243852 (PMC7745967; doi:10.1371/journal.pone.0243852)
Supplement: S1 Appendix — (PDF) [file pone.0243852.s001.pdf]

S1 Appendix. Systematic Mapping Study Resulting Catalog

Table 1. Catalog of papers that propose associations between software development and SO.

ID	RQ1: Association Strategies	RQ2: Input	RQ2: Output	RQ3: Evaluation	RQ4: Results
S1	Hipikat infers links by combining information contained within the project artifacts and the meta-information about the artifacts from different information sources	Artifacts (tasks, source file versions, messages on forums, project documents) produced during development	Artifacts	Qualitative. Newcomers using Hipikat x experienced developers	Interview: Newcomers found related files faster, although experienced users performed better in easy and difficult tasks.
S2	Uses the source code to generate SO queries. Manual, difficulty, user action, error.	Text similarity between Code or Exception on IDE	SO code snippets or posts	Qualitative. Log analysis of tool's performance. Clicking in a query's result indicate effectiveness.	794 queries logged. 53% manual, 19%difficulty, 15% user action, 13% error.
S3	Associate text of APItutorial with SO using CosineSimilarity	API Tutorial	SO post	Quantitative. 30 SO posts for each API: JodaTime, Math, Official Collections, Jenkov Collections Smack APIs	Precision: Top5: 24.32% Top10:20.67% Top15:19.18% MRR:34.42%.
S4	Text Similarity between issue's text and SO posts text	Issues from cookbooks for Swing, Boost and LINQ	SO post	Quantitative. 35 cookbook tasks were submitted to a SO dump (similarity). Results were manually analyzed by authors qualitatively.	NDCG: 0.3583 (35%) for Relevance 0.5243 (52%) for Reproducibility
S5	Text Similarity between issue's text and SO posts text	Android's Issue text	SO post	Quantitative. Uses Android Issue Tracker Tests Semantic Similarity w/ Stack Dump, Temporal Similarity and, Temporal and Internal Links.	Precision:Top10: 0.49, 0.56, 0.62.MRR: 0.29, 0.33, 0.37.

S6	Text Similarity between IDE exceptions text and SO posts text	IDE exception's errors text	SO post and search queries	Builds a SO corpus context (exception / code). 38 unique traces from6 grad students. 37 common exceptions from Java were included.	MeanPrecision: Top10:0.1229 Top20:0.0736 Top30:0.0538 Accuracy: Top10: 68% Top20:73.33% Top30:74.66%
S7	Text Similarity between issue's text and SO posts text	Android's Issue text	SO post	Quantitative. Compare Issue's Title with SO's Title. Used 2 Samples. Compare SO's return with SO's links on issue trackers.	Precision: Top10:33:16% Top20:36:88% Top100: 47:27%
S8	Suggests SO code snippets based on the context inside Eclipse	Code	SO post (Snippets)	Quantitative. 3 experiments with 35 Java Exercises from a book.	NDCG: 0.0907 (9,07%) for Relevance. Precision: Top10:0.0243 Top20:0.0135 Top30:0.0099 Accuracy: Top10:18.92% Top20:18.92% Top30:18.92% [17]
S9	Harnessing SO for the IDE	Same as S8			
S10	Support API designers to find expert information on Q&A websites. Propose (LDA) to reduce the dimensionality of SO posts, statistical techniques to mine SO posts and metrics to rank SO posts.	Textual data (questions and answers) and meta-data (such as "posted-by", "posted-time", and "'number-of-views").	SO Posts	Compare approach with two baselines. Case study on posts related to the APIs of Apple's iOS and Google's Android. Manual validation of 1,200 posts.	Up to 93% accuracy, 34% less noise, (i.e., how-to and implementation-specific questions) when compared to baselines.

S11	When an exception occurs, analyzes the stack trace to locate the program statements that caused the exception and presents candidate solutions to the exception, including code snippets and developers' discussions.	Generate a textual query combining exception + contextual information. Search in SO uses REST interface.	SO Posts	No formal evaluation presented.	No formal evaluation presented.
S12	Analyzes code context in the IDE, searching for SO posts; considers aspects such as code aspects (e.g., code clones, type matching), conceptual aspects (e.g., textual similarity), and community aspects (e.g., user reputation).	Code	SO Posts	One study evaluated qualitatively while another evaluated the model [10]	
S13	Tool translates the query into list of relevant API classes, mining keyword-API associations from SO, and applies the reformulated query to GitHub code search API.	Accepts an unstructured natural language query (i.e., does not require API information).	Code snippets	Conduct experiments using 150 code search queries randomly chosen from three programming tutorial sites–KodeJava, Java2s and JavaDB.	Tool was able to suggest at least one relevant API class for 79% of the queries within the Top-10 API suggestions.

S14	The semantic similarity between bugs and Question and Answer (Q&A) posts posted on OSS projects is analyzed by integrating the contents of the repositories based on text mining approach.	OSS Bugs Text	SO Posts	Integrates bug tracking system and SO, linking bugs to related Q&A posts (if possible) based on text mining approach and comparing the average bug fix time of posted and non-posted bugs. Temporal features and cosine similarity used.	Non-posted bugs had a higher days to fix average, i.e., found that it will take less time to fix the bugs if it is posted in SO.
S15	Use LDA model and Q&A meta-information to automatically generate query from code context and recommend the retrieval Q&A to developers	Code	SO Posts	Authors collected a code test data by themselves and tested using a baseline and compared to their proposed LDA model.	Average precision of 41%.
S16	Trained model captures similarity between bugs and corresponding useful forum threads, and thus recommend useful threads for a newly reported bug.	Bug text	SO Posts	Extracts 2,000 pairs of GitHub issues and SO threads and identifies the semantic similarity between bugs and threads.	Precision: 0.6043 / MRR: 0.3832 / Recall: 78.50% for Top10
S17	Automatic approach which integrates internal citations, semantic similarity with temporal factors to mine potential associations between them for automatic knowledge sharing between Android Issues and SO	Issues from Android	SO Posts	Cluster posts based on the semantic similarities and components diameters. Similarity threshold as 0.3.	Precision 62.51% for Top10

S18	Automated approach to find API classnames that are semantically related to a given natural-language query	Natural language query	SO Posts	74 queries on a corpus of 23,677,216 code snippets that are extracted from 24,666 open source Java projects. Apply bag-of-words model, compute semantic distance between the initial query and API class-names and select most related API class-names	For 10 retrieved results for 76% retrieves relevant examples
S19	Associates tasks by their similarity using Leveishtein similarity algorithm	Similar tasks	SO Posts	Qualitative study asks developers if similar tasks found could reuse SO posts originally used in base tasks	30% of tasks could have posts reused
S20	Same as S6				
S21	Developers can use natural language to describe the Java programming tasks as a query.	Natural language query to be expanded	SO Posts (Java API Methods)	Manually selected 413 Java-API-related questions from SO and labeled the ground-truth APIs for these questions based on their accepted answers.	Tool outperforms baselines by at least 42% in terms of MAP and MRR.

S22	Computes similarity between the bug report and post of SO by combining two similarity measures, namely Cosine and Okapi.	Bug Report	SO Posts	Create datasets for evaluation from open source projects that are Aspectj, Birt, Eclipse platform UI, JDT, SWT, and Tomcat. Randomly selects 100 bug reports from these projects to test model. To measure the performance of the proposed model.	AspectJ project bug report: MAP: 0.41 and MRR: 0.83
S23	Same as S6				
S24	Proposes a question-code matching model so developers can find relevant code to their questions on SO.	Queries	SO Post Code	Implemented model with trained data from SO.	MRR: 0.38. Accuracy: Top5-0.57/Top3:0.44 Top1:0.20
S25	Propose automated techniques to mine API usage scenarios from SO combining a code example, the task description, and the reactions of developers towards the code example.	Code + Task description + developer reaction	SO Post (API usage scenarios)	Compare algorithm with 7 baselines and performs a user study of 31 software developers.	Result shows that the participants preferred the mined usage scenarios in Opiner over API official documentation.
S26	Associates tasks with SO Posts, by combining lexical descriptions of APIs with the task description. CROKAGE tool. Extends BIKER by not only suggesting a limited set of APIs.	Tasks	SO Posts	97 programming queries, using 50% to train and 50% to test and a user study with 24 developers.	79% Top-10 Accuracy, 40% precision, 19% recall, and a reciprocal rank of 0.46. Developers find recommendation relevant.

S27 (SB)	Web search interface integrated into the Adobe Flex Builder dev environment, helps users locate example code, augmenting query with context from IDE	Code + context	Code examples from web	Qualitative study with 20 participants	Participants found and adapted example code significantly faster; wrote significantly better code.
S28 (SB)	Eclipse plug-in that monitors the occurrence of exception stack traces in the Console View and detect the information need of the developer.	Exception text	SO Posts	Randomly collected 30 questions posted on SO and queries these posts with context, keyword and on SO directly.	Results show approach with context proposed performs better on top20 SO Posts, with avg rank of 3.9 and 20 matches.
S29 (SB)	Same as S4				
**A**	**Suggests SO Posts of similar project tasks**	**Project Tasks Similarity**	**Curated SO Posts**	**Quantitative. Dataset from industry used in model implementation.**	**Precision: 71.60%. Accuracy: 77.78% (Jaccard Similarity)**
